# Neuromusculoskeletal Simulation Reveals Abnormal Rectus Femoris-Gluteus Medius Coupling in Post-stroke Gait

**DOI:** 10.3389/fneur.2019.00301

**Published:** 2019-04-02

**Authors:** Tunc Akbas, Richard R. Neptune, James Sulzer

**Affiliations:** Walker Department of Mechanical Engineering, University of Texas at Austin, Austin, TX, United States

**Keywords:** neuromusculoskeletal simulation, stroke, stiff-knee gait, abnormal coordination, exoskeleton

## Abstract

Post-stroke gait is often accompanied by muscle impairments that result in adaptations such as hip circumduction to compensate for lack of knee flexion. Our previous work robotically enhanced knee flexion in individuals post-stroke with Stiff-Knee Gait (SKG), however, this resulted in greater circumduction, suggesting the existence of abnormal coordination in SKG. The purpose of this work is to investigate two possible mechanisms of the abnormal coordination: (1) a reflex coupling between stretched quadriceps and abductors, and (2) a coupling between volitionally activated knee flexors and abductors. We used previously collected kinematic, kinetic and EMG measures from nine participants with chronic stroke and five healthy controls during walking with and without the applied knee flexion torque perturbations in the pre-swing phase of gait in the neuromusculoskeletal simulation. The measured muscle activity was supplemented by simulated muscle activations to estimate the muscle states of the quadriceps, hamstrings and hip abductors. We used linear mixed models to investigate two hypotheses: (H1) association between quadriceps and abductor activation during an involuntary period (reflex latency) following the perturbation and (H2) association between hamstrings and abductor activation after the perturbation was removed. We observed significantly higher rectus femoris (RF) activation in stroke participants compared to healthy controls within the involuntary response period following the perturbation based on both measured (H1, *p* < 0.001) and simulated (H1, *p* = 0.022) activity. Simulated RF and gluteus medius (GMed) activations were correlated only in those with SKG, which was significantly higher compared to healthy controls (H1, *p* = 0.030). There was no evidence of synergistic coupling between any combination of hamstrings and hip abductors (H2, *p* > 0.05) when the perturbation was removed. The RF-GMed coupling suggests an underlying abnormal coordination pattern in post-stroke SKG, likely reflexive in origin. These results challenge earlier assumptions that hip circumduction in stroke is simply a kinematic adaptation due to reduced toe clearance. Instead, abnormal coordination may underlie circumduction, illustrating the deleterious role of abnormal coordination in post-stroke gait.

## Introduction

Following a stroke, individuals often experience significant impairments including muscle weakness, spasticity, increased tone, and abnormal coordination (1), which often results in compensatory movements (2). Abnormal coordination has been quantified in the upper limb (3), but only more recently in the lower limbs using joint torque measures (4–7), mechanical perturbations (7, 8) and H-reflex stimulations (9–12). However, it is unclear whether such abnormal coordination has direct consequences on gait function. Descriptive gait analyses based on biomechanical data and simulated muscle activations during gait in post-stroke individuals (13–15) and gait in cerebral palsy (16, 17) suggest that lack of lower limb coordination is related to gait dysfunction. Yet descriptive analysis lacks the ability to identify the mechanisms of abnormal coordination that perturbation methods can provide.

Our previous work developed a device that delivers controlled knee flexion torque perturbations during gait (18) and applied it to individuals post-stroke with Stiff-Knee Gait (SKG) (19). SKG is defined by reduced knee flexion during the swing phase, often assumed to be compensated for with hip circumduction (2). However, when exposed to pre-swing knee flexion torque perturbations, those with SKG walked with exaggerated hip abduction during swing instead of the expected reduction, while there was no change in healthy controls (19). Biomechanical factors such as balance and perturbation dynamics could not account for the increased abduction. These results suggested that hip abduction may not be solely acting as a compensatory motion for reduced knee flexion. We examined this possibility further by restricting knee flexion in healthy individuals with a knee brace (20). We observed that hip hiking, not abduction, compensated for reduced knee flexion, despite persistently high hip abduction in those with post-stroke SKG. Thus, abnormal neural behavior appears to underlie the hip abduction in people with SKG. However, the neural mechanisms leading to the exaggerated hip abduction remain unclear. The abnormal cross-planar kinematics were likely the result of abnormal heteronymous muscular activation initiated via reflexive (8) or voluntary (4, 21) mechanisms. For instance, cross-planar reflexive couplings between adductor longus (AL) and RF were observed in individuals post-stroke while in a seated position (8). Also in post-stroke individuals, voluntary activation of the hamstrings resulted in a cross-planar coupling with adductors while standing (4). This is in agreement with previous simulation analyses of post-stroke gait showing synergetic coupling between hip abductors and knee flexors in the swing phase (22). Based on this information, the exaggerated abduction observed in our previous work may have been the result of a reflexive stimulus of the RF coupled with abductor muscles (Hypothesis 1). In contrast, such abductor activity may have been coupled with voluntary initiation of the hamstrings (Hypothesis 2) as an adaptation to the perturbations.

In this work, we seek to delineate the underlying muscle activation behind the abnormal cross-planar kinematics. We used neuromusculoskeletal modeling and simulation (NMMS) to derive the estimated muscle states and supplement measured EMG. NMMS combines the use of a lower limb musculoskeletal model with measured kinematic and kinetic data to estimate muscle states (23, 24). Descriptive NMMS simulations have identified the role of rectus femoris in post-stroke gait (25). Here we used NMMS to estimate muscle states that are difficult to measure experimentally during gait, such as fiber stretch velocity and all lower limb muscle activities, including abductor muscles that are difficult to access using EMG. The accuracy of the simulated fiber stretch measures were obtained from Hill-type muscle models verified by muscle moment-arms of cadaver subjects (26–28). Simulated fiber length and velocity has been used for generating tunable models for contracture and spasticity assessments (29) and determining operating range of fibers during walking with different speeds (30, 31). Thus, together with measured EMG, NMMS can help elucidate mechanisms of cross-planar muscle synergies and abnormal reflexive responses. Evidence of an abnormal reflex coupling underlying excessive hip abduction during knee flexion perturbations in those with SKG would manifest itself as a hyperactive RF stretch reflex followed by heteronymous activation in the hip abductors (H1). Alternatively, if the abnormal coupling was generated by the voluntary knee flexion movement and due to lack of independent joint control, temporary removal of torque perturbations (“catch trials”) should result in correlated activation between the hamstrings and abductors (H2). This study represents a novel approach toward delineating the differential roles of impairments in post-stroke gait.

## Methods

### Experimental Data

Nine chronic, hemiparetic participants with post-stroke SKG and five healthy, unimpaired individuals gave written informed consent using procedures approved by the local Institutional Review Board to participate in the experiment and described in detail in previous work (19). Inclusion criteria for the hemiparetic participants included reduced knee flexion angle during swing and the ability to walk for 20 min without rest at 0.55 m/s on a treadmill (19). A lightweight, custom-designed powered knee orthosis was used to provide knee flexion torque perturbations during the pre-swing phase without affecting the remainder of the gait cycle (18). The level of torque perturbations was automatically adjusted to maximize the swing phase knee flexion angle in those with SKG, whereas in healthy controls the torque was adjusted to increase knee flexion angle at 20° during the swing for each subject prior to commencing the experiment.

The protocol consisted of 610 steps and lasted 16 min. No perturbation was applied during the initial 50 steps (baseline) and the perturbation was applied for the next 560 steps (perturbed). Also, four non-consecutive trials (catch trials) with no perturbation during a single gait cycle were implemented throughout the perturbation period. Lower limb kinematic data were collected using optical motion capture (Motion Analysis, Santa Rosa, CA), ground reaction forces were collected using an instrumented treadmill (Tecmachine, Andrez Boutheon, France), and the applied perturbation torque was collected through the knee orthosis. Surface EMG (Delsys Inc., Boston, MA) data were collected at the muscle belly of RF, vastus lateralis, adductor longus, medial hamstrings in healthy participants and additionally the tensor fascie latae (TFL), and vastus medialis in hemiparetic individuals. Motion capture data were collected at 120 Hz, whereas other data were collected at 1 kHz. Details of experimental procedure are described in Sulzer et al. (19).

### EMG Analysis

EMG data were used to determine reflex activation and validate the NMMS model. All signal processing of EMG data was performed in MATLAB (MathWorks, Natick, MA). To evaluate reflex activation, each EMG signal was demeaned, rectified and then integrated over a temporal window 100 ms prior to toe-off, which was the window expected to reveal stretch reflex response to perturbations (32). We analyzed 10 and 20 consecutive steps from baseline and perturbation periods, respectively. For NMMS validation, the EMG signals were demeaned, rectified and low-pass filtered with a 5th order Butterworth filter at 20 Hz. The processed signals were then synched with kinematic data, divided into gait cycles and integrated into 1% gait cycle intervals to obtain integrated EMG (iEMG) measures. The magnitude of iEMG was normalized to the participant's own maximum overall iEMG across all trials.

### Neuromusculoskeletal Model and Simulation

**Figure 1:**
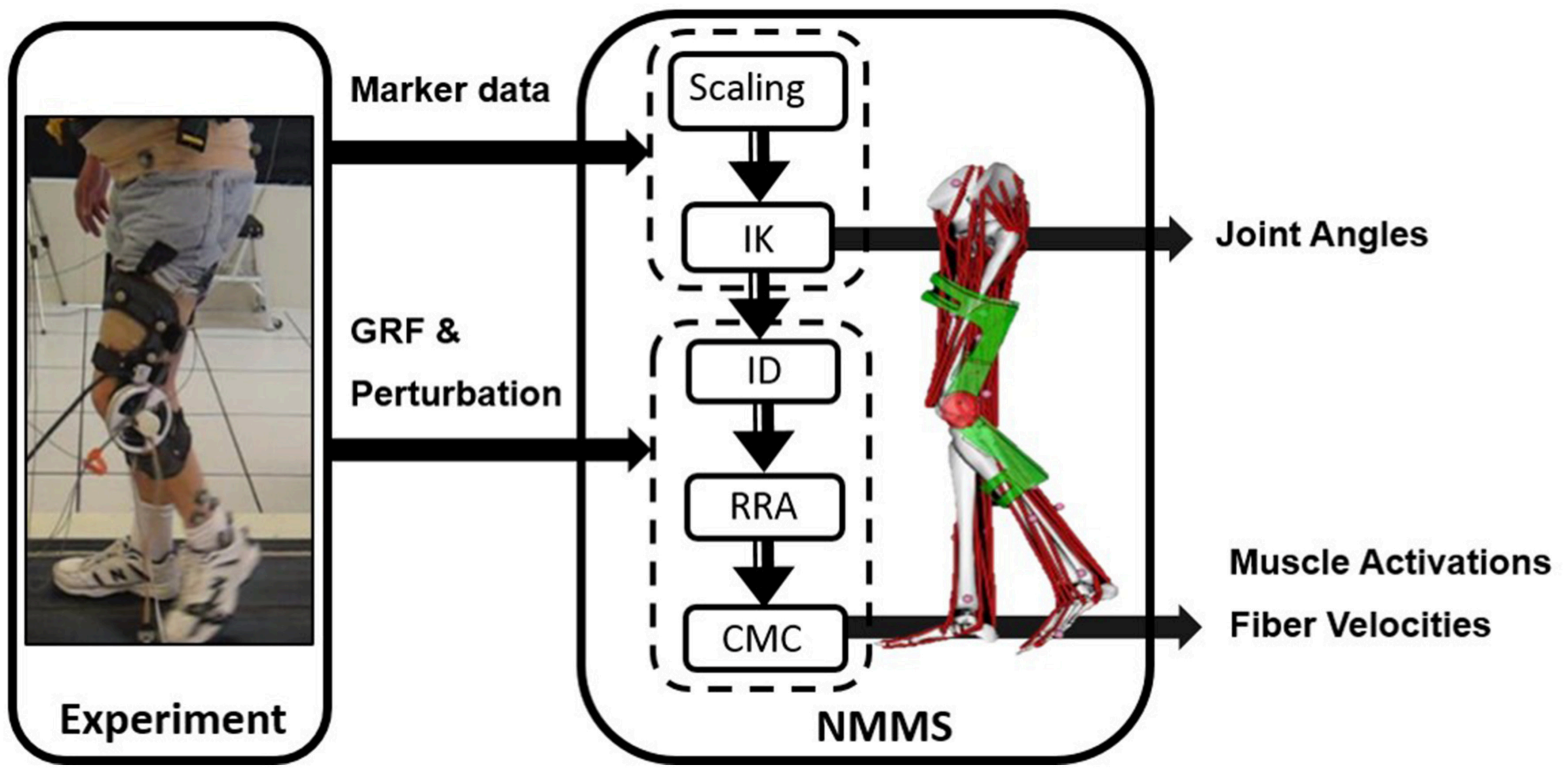
Neuromusculoskeletal modeling and simulation (NMMS) framework. Experimental data is used as input to determine joint angles, and muscle length, velocity and activation profiles. The NMMS processes are shown in the solid frame on right. Height adjusted models are generated for each participant using the scaling tool and joint angles are calculated using inverse kinematics with the least square fit for marker trajectories. The muscle activation and fiber velocity profiles are determined using computed muscle control (CMC), and validated with the experimentally measured EMG data.

We used NMMS to determine muscle states from kinematic, kinetic, and perturbation data with the Gait 2392 musculoskeletal model of OpenSim version 3.3 (33). The model consisted of 18 degrees-of-freedom and 80 muscles. We condensed the head, arms and trunk (HAT) to the pelvis segment in the model since no markers were placed on the HAT. For each participant, the joint angles were calculated using inverse kinematics with the least square fit of marker trajectories. The residual reduction algorithm (RRA) was used to fine-tune the model to minimize the residuals (artificial forces and moments to maintain dynamic consistency of the simulation) between the kinematic and GRF data. The parameters required to test our hypotheses, i.e., muscle activation profiles and muscle fiber stretch values, were calculated using computed muscle control (CMC). CMC identifies the muscle activation patterns to produce a forward dynamics simulation emulating the experimentally collected data. Figure 1 illustrates the NMMS procedure.

The knee flexion perturbation induces opposing torques on the shank and thigh (18), which were modeled as coupled forces on the tibia and femur in OpenSim. The coupled forces were oriented in opposite directions and set equidistant from the center of mass of the corresponding bodies. We validated the accuracy of the perturbation by comparing the simulated torque at the knee with and without the modeled perturbation to the measured torque from the device. In addition to validating knee flexion perturbation values, we also accounted for unmeasured handrail forces using estimation procedures previously described (34). Some adjustments and constraints were made to the model in order to more accurately represent the experimental conditions. For instance, in the experiment, the left thigh markers were attached directly to the device. The compliance of the interface between the brace and the leg reduced the accuracy of the measured knee flexion angle, thus we reduced the weightings of those markers accordingly. The dynamic consistency of the simulations was evaluated by examining the resulting residual forces and moments (Table S2) and verifying them with the OpenSim guidelines (35).

We used CMC to generate muscle activations and muscle fiber stretch velocities from the muscle-driven simulation based on the subject's movement. To validate the simulated muscle activations from CMC, we used the EMG data collected from RF, AL, VL, and semimembranosus (SM). We qualitatively compared the estimated muscle activities and iEMG for both the healthy controls and those with SKG with and without perturbation. We confirmed the activations in the model occurred during the appropriate time in the gait cycle by comparing the simulated muscle activations with the measured iEMG activity on each participant (Figure S1).

Our primary hypothesis (H1) was that the exaggerated abduction in those with SKG occurs simultaneously with a hypersensitive quadriceps stretch reflex. A reflex would occur within 120 ms of the stimulus considering both mono- and polysynaptic mechanisms (36). This time interval has been implemented for detecting reflexive responses from quadriceps following mechanical perturbation during gait (32). We defined the stimulus onset, the initiation of the involuntary response, as the timing of the peak stretch velocity. We calculated the stretch velocity of the quadriceps muscle group (RF, VL, VM, and vastus intermedius: VI) by taking the time derivatives of the fiber length measures obtained from the simulation. Then we evaluated all the estimated muscle activities during the involuntary response (IR) period within 120 ms after stimulus onset indicated by simulated peak fiber stretch velocity, integrated over a ±1% gait cycle window around the peak. Correspondingly, the voluntary muscle response (VR) was evaluated within the voluntary time window, 120–300 ms following the stimulus onset.

It is also feasible that a previously unaddressed excessive RF activity alone could produce sufficient abduction motion during gait since the muscle has a small abduction moment arm. To test this possibility, we used the musculoskeletal model and modified the RF activation to the maximum value (100%) between the pre-swing and mid-swing phases. We also implemented a partial forward dynamics simulation of a representative stroke subject with the applied perturbation. We forced the simulation to follow the motions calculated from the experimental data for all joints except hip abduction of the affected side, which is driven by the simulated muscle activity patterns. We compared three different cases: (1) simulated abductor and RF activations from perturbed gait, (2) simulated abductor and RF activities from baseline, and (3) simulated abductor activity from baseline and full RF activation (100%).

The alternative hypothesis (H2) was the voluntary knee flexion-hip abduction coupling originating from the lack of independent joint control (abnormal synergies), exaggerated as an adaptation to the perturbations. The specific mechanism could be a coupling between hamstrings and abductors (4). In this case, we focused on a different set of steps when the perturbation was temporarily removed (catch trials), expecting a coupling between abductor and hamstring activity dependent on gait adaptation (37). To test this, we integrated simulated hamstrings activities, including semimembranosus (SM), semitendinosus (ST), and biceps femoris long head (BFlh) within the peak abductor activation regions (±2% gait cycle) for the baseline and catch trials.

### Statistical Analysis

The hypothesized reflex coupling (H1) requires a coupling of the homonymous (i.e., quadriceps) and heteronymous (i.e., abductor) muscle activation (8) within a reflex latency following the perturbation. We established activity of quadriceps during this IR period both experimentally and through simulation. Experimental evidence of quadriceps activity during the IR period was provided by quadriceps iEMG within 120 ms following stimulus onset. We used a 2 × 2 repeated-measures analysis of variance (ANOVA) (α < 0.05) with two factors (group and perturbation), each with two levels (healthy controls and SKG, perturbation and baseline) followed by Tukey-Kramer *post hoc* testing to compare quadriceps iEMG between groups. We predicted a significant interaction effect between group and condition, with RF iEMG being higher in those with SKG compared to the healthy controls. Simulation was also used to estimate quadriceps activity during IR period. We implemented a linear mixed-effects model, taking groups and fiber stretch velocities as covariates, and dependent variables of simulated quadriceps (RF, VL, VM, and VI) activations. We predicted a significant correlation between simulated RF activation in the IR period and its fiber stretch velocity in those with SKG (α < 0.05). Lastly, the coupling between quadriceps and abductor activity was investigated using a linear mixed model with peak simulated quadriceps activation as the independent variable and abductor muscles [GMed, GMax, GMin, TFL, sartorius (SAR), and piriformis (PIR)] as dependent variables during IR period. We predicted a significant correlation between RF and abductor activity in SKG compared to the healthy controls.

A voluntary abnormal coordination pattern (H2) would be represented by coupled activation regardless of perturbation. A linear mixed-effects analysis was used with factors of condition (catch trials and baseline) and groups (healthy and SKG). We examined all combinations of simulated hamstrings activity occurring simultaneously with peak simulated abductor muscle activations (GMax, GMin, GMed, and TFL) in the VR period as dependent variables. We expected to find a significant correlation between hamstring and abductor activation in those with SKG compared to the healthy controls. For all linear mixed-effect models subjects were included as random variables. All statistical analyses were performed using R software (38) using a linear mixed model (lme4) package.

We implemented the Shapiro-Wilk normality test for the quadriceps iEMG measures and primary simulated measures (simulated RF activation and RF fiber stretch velocities) for each participant for baseline and perturbation condition (*p* > 0.05). We used Cohen's *d* to calculate the effect size between conditions for iEMG measures and for the significant interaction effect between group and conditions in linear mixed-effect models (39).

## Results

### RF Activity in IR Period (H1)

**Figure 2:**
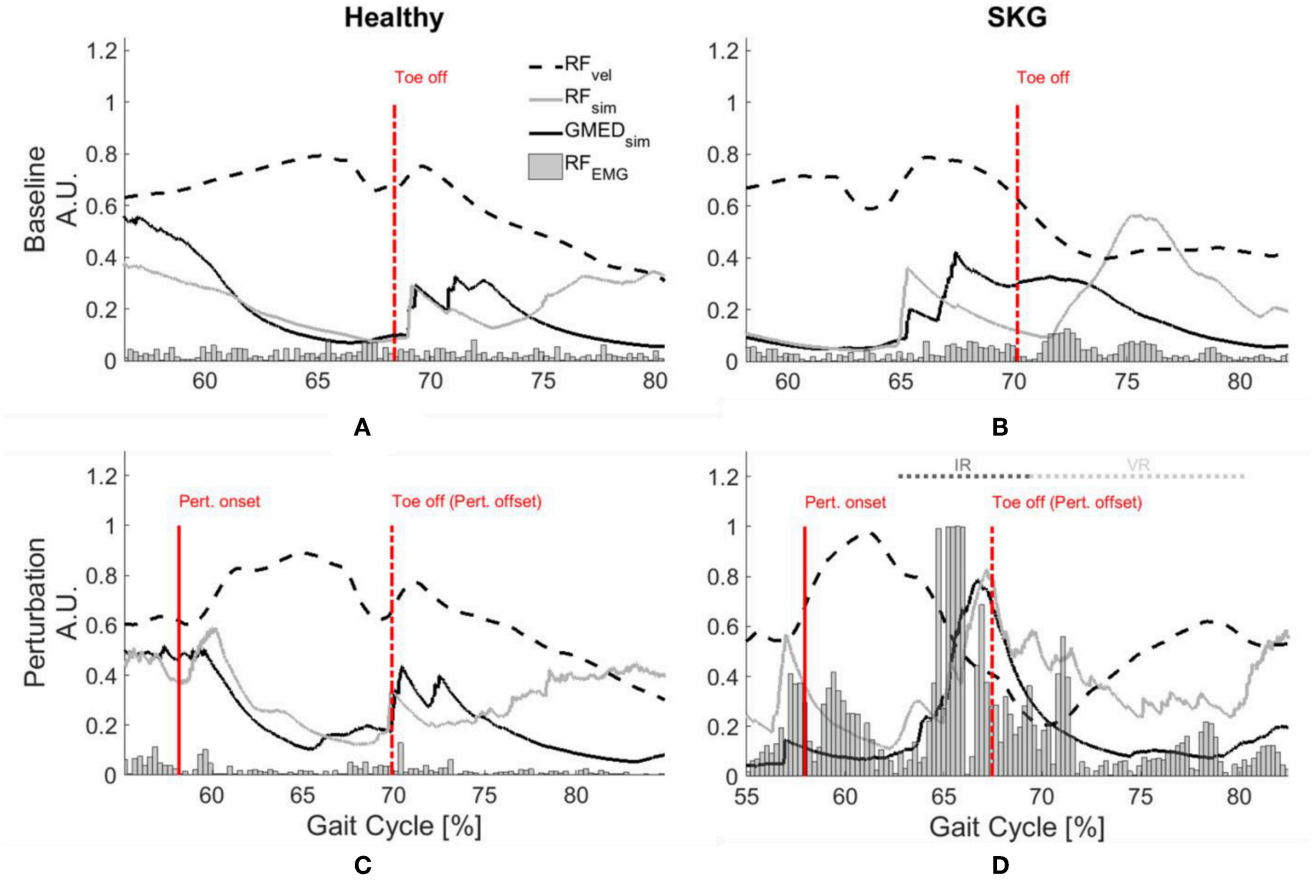
GMed and RF activations are increased with RF stretch velocity in stroke. Rectified RF EMG, simulated RF fiber stretch velocity and muscle activations of RF and GMed for a representative healthy control (left) and individual with SKG (right) during baseline **(A,B)** and with perturbation **(C,D)**. Each gait interval begins in pre-swing (55–60%gait cycle) and continues until late-swing (80–85% gait cycle). The RF fiber velocities were normalized with respect to the maximum of the demonstrated steps and RF EMG measures are normalized with respect the maximum values throughout the gait cycles of the corresponding subjects. The RF fiber stretch velocity values are increased for both healthy and SKG individuals with the perturbation. However, the raw RF EMG measures and simulated GMed and RF activity only increased in the individual with SKG following peak RF fiber velocity. This is indicative of an abnormal coupling pattern between RF and GMed in post-stroke SKG. The activations of RF and GMed are increased during the involuntary response (IR) period (<120 ms).

**Figure 3:**
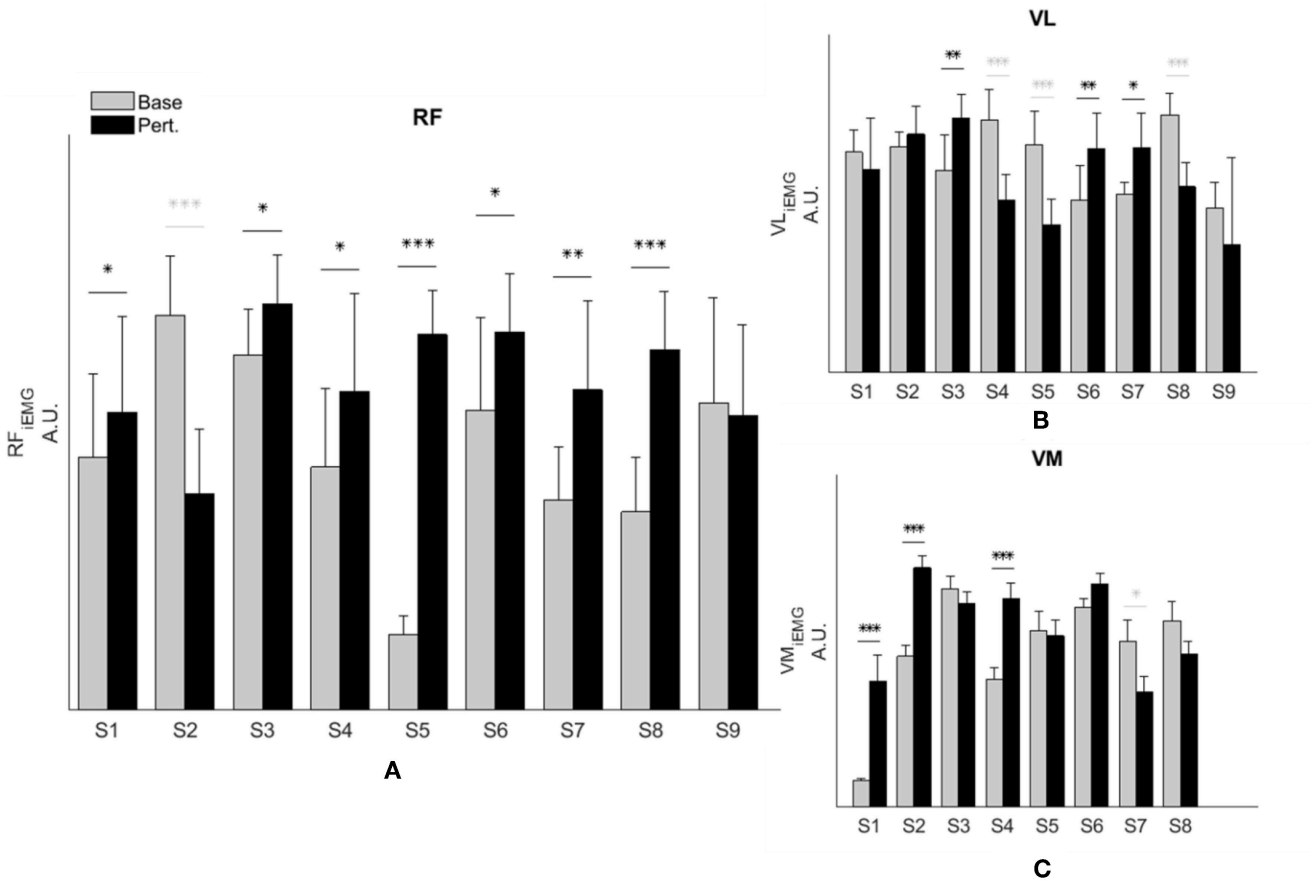
RF activity increases during knee flexion perturbation consistently in people with SKG. RF iEMG **(A)** and VM iEMG **(B)** values were increased significantly within the SKG group between baseline and perturbation (*p* < 0.001, *p* < 0.005 respectively). No significant changes were observed in VL iEMG **(C)** values (*p* = 0.06) based on the repeated measures ANOVA. The increase was more consistent for RF iEMG values across subjects compare to VM iEMG values. “Base” refers to unperturbed Baseline steps, whereas “Pert.” refers to steps with torque perturbations during pre-swing. Significant increase (black) and decrease (light gray) within subjects between baseline to perturbation conditions was determined using Tukey-Kramer *post-hoc* testing (^*^p < 0.05, ^**^p < 0.01, ^***^p < 0.001).

Representative data show increased simulated RF activations and EMG measures in response to the perturbation of an impaired individual, with no such effect on the healthy control (Figures 2A–D). These effects were similar at the group level. We observed a significant interaction effect across groups (healthy controls and SKG) and conditions (baseline and perturbation) in RF iEMG [*F*_(1, 321)_ = *15.5, p* < *0.001*] and VM iEMG [*F*_(1, 321)_ = *2.42, p* < *0.002*] but no significant interaction was observed for VL iEMG [*F*_(1, 321)_ = *3.79, p* = *0.06*]. Between knee perturbations and baseline trials, RF iEMG increased [*F*_(1, 235)_ = *86, p* < *0.001, d* = *0.42*]. The increased RF activity was consistent across subjects (Figure 3A). Similarly, there was an increase in VM iEMG [*F*_(1, 235)_ = *8.01, p* = *0.005, d* = *0.51*] but this increase was not consistent across subjects (Figure 3B). For healthy controls, we found a decrease in VL iEMG [*F*_(1, 86)_ = *9.04, p* = *0.008, d* = −*0.57*] whereas no significant changes were observed in VM iEMG [*F*_(1, 86)_ = *0.26, p* = *0.614*, Figure 3C] or RF iEMG [*F*_(1, 86)_ = *0.02, p* = *0.89*].

**Figure 4:**
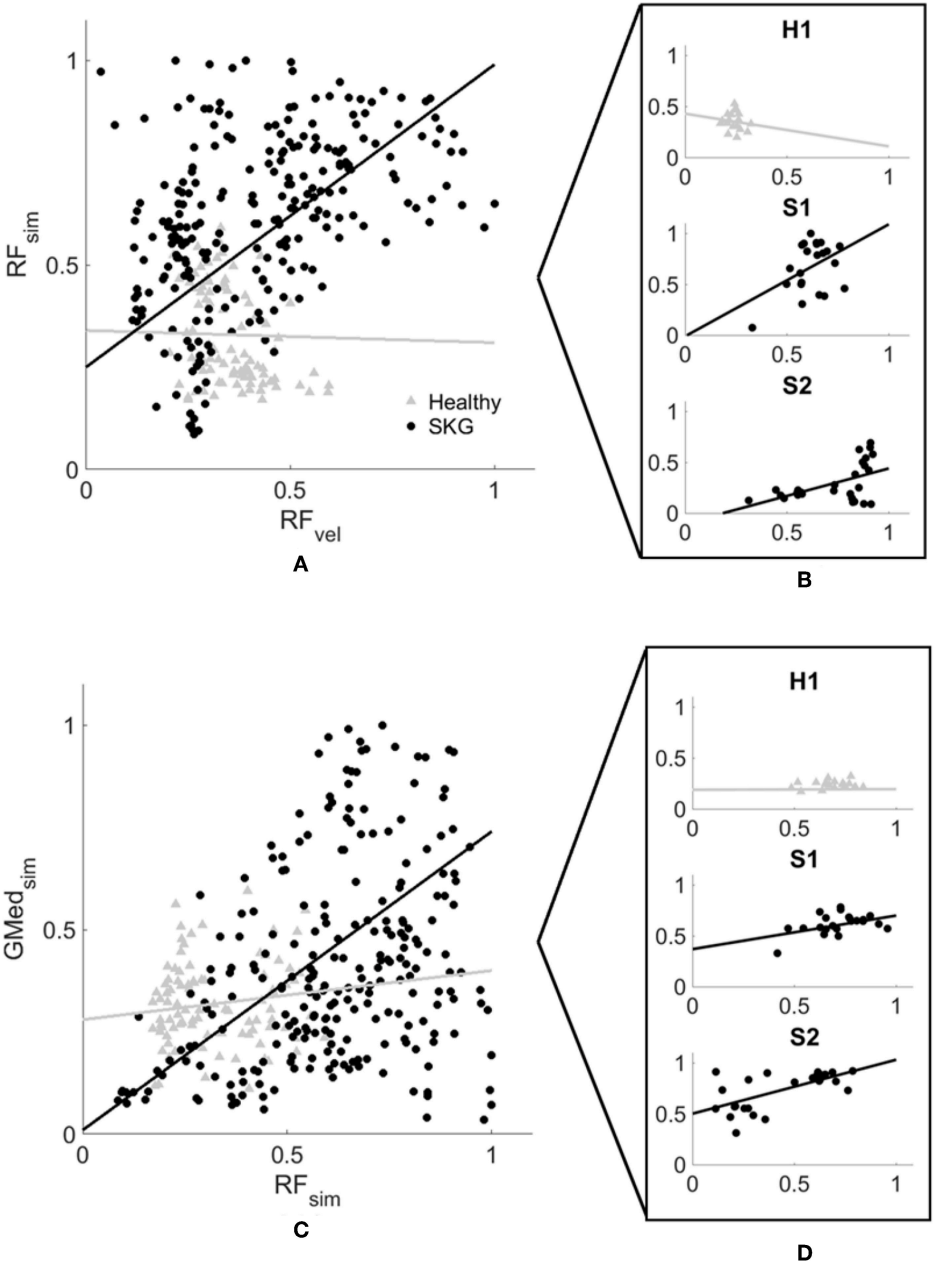
Computed RF activation increases with RF_vel_ **(A,B)** and coupled with GMed **(C,D)** activity in people with SKG. Normalized RF peak stretch velocity (RF_vel_) values and peak RF activation **(A)** and coupled GMed activity **(C)** from every simulated gait cycle in all subjects during the involuntary response period. Individuals with SKG (dark circles) show increased peak RF (β_SKG_ = 0.8) activity and coupled GMed (β_SKG_ = 0.73) activity, as would be expected in a reflex response. However, healthy controls (gray triangles) show a decrease in RF (β_Healthy_ = −0.03) and small increase in coupled GMed (β_Healthy_ = 0.12) activity, suggesting that this abnormal reflexive response did not exist in healthy controls. A representative healthy participant (H1) and two representative subjects with post-stroke SKG (S1, S2) highlight the correlation between RF_vel_ and RF activation profiles **(B)** and coupled GMed activity **(D)**.

Simulation results were consistent with EMG measures following the perturbation. Figure S1 shows general agreement between measured and computed muscle activations. We also verified the dynamic consistency of the simulation with the experimental measures kinematic and kinetic and accuracy of the simulated muscle actuation with residual forces and moments from RRA tool and reserved actuators from CMC tool (Table S2) according to the guidelines of OpenSim (35). Figure 4 shows every modeled step from all subjects and subsequent regression lines derived from the linear mixed model. Figure 4A illustrates peak modeled RF activation following increased RF fiber stretch velocity (RF_vel_) during the IR period; RF activation increased with RF_vel_ in the SKG group (β_SKG_ = 0.8) but slightly decreased in the healthy controls (β_Healthy_ = −0.03). Compared to healthy controls, people with post-stroke SKG had a greater correlation between simulated RF_vel_ and RF activation [*F*_(1, 321)_ = *30.2, p* = *0.001, d* = *0.63*, Figure 4A). The effect was consistent across SKG subjects (Table S1).

We found no significant difference between correlations during the voluntary response period [*F*_(1, 321)_ = *0.59, p* = *0.443*]. We did not find a significant correlation between the activation of other quadriceps muscles during the IR period with increased RF_vel_ [VL: *F*_(1, 321)_ = *0.31, p* = *0.57*, VM: *F*_(1, 321)_ = *0.66, p* = *0.412*, VI: *F*_(1, 321)_ = *2.81, p* = *0.092*]. The increased RF activation applied to the generic Gait 2392 musculoskeletal model resulted in < 2° increase in hip abduction, which was less than the average increase (5°) observed in those with post-stroke SKG. The result of forward simulation with full RF activation in the representative subject with post-stroke SKG resulted in greater hip abduction than baseline (3°), which was 3.5° lower than the abduction following the mechanical perturbation (7.5°).

### Reflex Coupling With Abductors (H1)

Representative data show no coupling between simulated RF and GMed activation in the unimpaired individual (Figures 2A,C). However, when perturbed, the individual with SKG exhibited co-activation of RF and GMed within the IR period (Figures 2B,D). At the group level, simulated GMed activation was correlated to peak RF activation [*F*_(1, 321)_ = *7.15, p* < *0.001, d* = *0.30*, Figure 4C] within the IR period in SKG subjects. This is further illustrated in Figure 4C for all steps and subjects, the correlation between GMed and RF activations were high in those with SKG (β_SKG_ = 0.73), but not for the healthy controls (β_Healthy_ = 0.12). The effect was consistent across seven of nine individuals with SKG (Table S1). We found significant correlations of GMax [*F*_(1, 321)_ = *5.90, p* = *0.015, d* = *0.28*] with RF activation in those with SKG, but not with other abductors, GMin [*F*_(1, 321)_ = *1.95, p* = *0.162*], TFL [*F*_(1, 321)_ = *3.02, p* = *0.083*], SAR [*F*_(1, 321)_ = *0.20, p* < *0.65*] or PIR [*F*_(1, 321)_ = *0.034, p* = *0.85*] (Table 1). There was no evidence of abductor activity in the VR period following peak RF_vel_ (*p* > 0.05).

**Table 1:** Couplings following perturbation in post-stroke gait.

**Muscle**	p_*x*1^*^x2_
GMed	0.0079^**^
GMax	0.015^*^
GMin	0.16
TFL	0.08
SAR	0.65
PIR	0.85

The table shows the level of significance (^*^p < 0.05, ^**^p < 0.01) between the corresponding simulated muscle activation profiles and peak RF activation within involuntary response period (x_1_) and given the fixed effects of groups (x_2_). The results indicate a significant increase between RF and gluteal muscle (GMed and GMax) activation profiles for individuals with SKG compared to the healthy controls during the involuntary response period. No significant difference found for other abductor muscles (GMin, TFL, SAR, and PIR) during this period.

### Voluntary Synergistic Coupling (H2)

Simultaneous activation of hamstrings and abductors could be part of an abnormal synergistic coupling. We found no significant differences in any combination of simulated hamstrings (SM, ST, and BFlh) and corresponding peak abductor (GMed, GMin, GMax, TFL, SAR, PIR) activations compared between conditions (catch trials and baseline) and groups (*p* > 0.05).

## Discussion

Previous research found exaggerated hip abduction in individuals with post-stroke SKG with applied knee flexion perturbations (19). We have proposed two possible abnormal neuromuscular mechanisms that could explain the increased hip abduction: a voluntary synergistic coupling between hip abduction and hip extension or an abnormal reflexive coupling between the quadriceps and abductor(s). The measured EMG and NMMS data both suggest that the flexion perturbation likely produced an abnormal stretch reflex in RF. This activity was coupled simultaneously with simulated GMed activation, but not the other non-gluteal abductors, suggestive of an abnormal RF-GMed reflex coupling (H1). This simulation reveals a novel hypothesis of RF-GMed coupling during post-stroke gait which further work can empirically validate.

These findings also challenge earlier assumptions of hip circumduction being part of a compensatory motion to account for reduced knee flexion (2). Our observations suggest that while motions such as vaulting and pelvic obliquity may compensate for lack of knee flexion, circumduction in SKG may be due to other factors such as abnormal coordination patterns. It should be noted that circumduction can be a necessary compensation for other disabilities such as foot drop, but for those with SKG, excessive plantarflexion is either non-existent or alleviated with an ankle-foot orthosis. Regardless of the level of ankle dysfunction in our participants, our data showed abnormal coordination between abductors and RF following knee perturbations. These abnormal patterns may be initiated by excessive RF activity, which is one of the suggested causes of SKG after stroke (25, 40–42). This initial evidence indicates that interventions intending to restore healthy, symmetric gait for those with SKG should consider hip circumduction as a discoordination pattern rather than a compensation. For example, hip abduction should be directly addressed as part of the impairment, such as by exercises that train the leg to move toward rather than away from the midline. Clinical interventions that stretch RF, including body-weight supported treadmill training (43) or robotic assistance (44) should consider that such training could excite abnormal coordination with hip abduction. Lastly, novel clinical interventions such as robotic exoskeletons could unintentionally exacerbate abnormal coordination patterns while attempting to support motion.

The simulated RF-GMed coupling appears to be unique. We found no evidence of increased EMG in the VL, VI or VM in those with SKG. However, we did find coupled simulated activation of the GMax in addition to GMed. We estimated the individual contributions of abductor muscles during the simulation to address the specificity of the abductor muscles involved in the coupling. For each abductor muscle, we removed the corresponding muscle from the model and allowed a reserved hip abductor/adductor actuator to replace the contribution of the moment generated by the removed muscle. The highest moment (>40 Nm) was generated by the reserved actuator after the GMed was removed followed by GMax with the second highest contribution (<10 Nm) indicating the essential role of GMed. These results suggest that GMed is the most likely contributor to excessive hip abduction observed in SKG with knee perturbations.

Our analysis shows a coupling between the RF and abductors in SKG, but not in healthy individuals. Further, this coupling appears to occur too soon after the perturbation to be attributed to a voluntary response. The neural mechanism underlying this coupling remains unclear. The malfunction in supraspinal control following stroke is suggested to cause impairment in the regulation of relevant spinal interneurons (45). This impairment could influence the medium latency stretch responses believed to be relayed through oligosynaptic pathways (46) which could initiate abnormal heteronymous stretch reflex responses in those post-stroke. Previous research has identified the existence of these abnormal reflex responses in static postures. For instance, increased soleus H-reflex excitations have been observed with simultaneous peroneal and femoral nerve stimuli suggesting the excitatory heteronymous pathways in stroke compared to healthy controls (11). Others have observed that mechanical perturbations of the AL resulted in a stretch reflex coupling with RF in individuals with chronic stroke (8). From our experimental data, we observed those with post-stroke SKG exhibit an increased RF EMG activity consistently within a latency following torque perturbation indicative of a hyperactive stretch reflex. This effect was not observed in EMG measures from VL and VM. The simulated RF activation was closely aligned with measured RF EMG activity as well as simulated simultaneous RF and GMed activation and consistent across subjects. The simulated vasti muscle activations were not aligned with abductor activations following perturbation indicating that the increased RF is initiating the abnormal involuntary response in abductors. Further experimental evidence is necessary to conclude the neural origin of this abnormal coordination pattern.

While NMMS provides rich information about the neuromusculoskeletal system, it has some limitations. Musculoskeletal modeling and simulation rely on a number of assumptions, including cadaver-based muscle moment arms (47), generic Hill-type muscle models and mathematically driven cost functions (48). The simulated fiber stretch length and velocity derived from Hill-type muscle models were able to predict estimated muscle forces using *in vivo* measures (49) and ultrasound imaging during dynamic movement (50). However, tuning of the muscle models based on muscle-specific (51) and subject-specific (52, 53) experimental measures and the relationship between force and intrinsic muscle properties (54) still require improvement for better accuracy. Since our aim was to characterize the neuromuscular mechanism behind the observed response using the simulation, we have not modified the neural control portion of the model with a stretch reflex controller (55) or models of spasticity (56, 57) to avoid manipulating the muscle states. As a result, simulations require validation with measured muscle activity. An alternative approach could be to use EMG-informed (58) or EMG-driven (59) simulations. Due to the inaccessibility of surface EMG for small abductor muscles and the limited number of total EMG measures in the experiment, we were not able to implement these approaches. However, our simulations were dynamically consistent with experimental force and position measures and were in good agreement with the EMG data of participants (Figure S1). In addition, the coupling between RF and GMed were consistent for the participants with post-stroke SKG (Figure 4, indicated by S1 and S2) with the highest and lowest correlation for simulated activations and RF EMG (Figure S1, indicated by S1 and S2). Furthermore, both simulated and experimental RF results showed consistent effects within and between subjects. Thus, our data suggest that simulated muscle activities are indeed realistic.

## Conclusion

Based on previous work (19), our simulation analysis shows that knee flexion perturbations in pre-swing initiate a response in RF in those with post-stroke SKG, indicative of hyperreflexia. Activity of RF coupled with simultaneously activated GMed suggests a previously unidentified heteronymous abnormal coordination pattern during post-stroke gait. Thus, hip circumduction may not be entirely a compensatory motion, but rather part of an abnormal coordination pattern. This information suggests that clinicians should treat excessive hip abduction together with quadriceps spasticity instead of encouraging the ostensible compensation. Our results also show how robotic exoskeletons may be effective at treating weakness, but could have side-effects on other impairments. Further work is needed to characterize the nature of this abnormal coordination pattern in stroke and its effects on function.

## Data Availability

The datasets for this manuscript are not publicly available because of confidentiality. Requests to access the datasets should be directed to James Sulzer, james.sulzer@austin.utexas.edu.

## Ethics Statement

The data was re-analyzed from previously published material and was fully anonymized thus exempt from ethics approval.

## Author Contributions

TA carried out simulation design, data analysis, statistical analysis, interpreted the data, and wrote the manuscript. RN contributed to simulation design, interpreted the data and critically reviewed the manuscript. JS was the principal investigator in this study and contributed to study design, statistical analysis, interpreted the data and critically reviewed the manuscript.

### Conflict of Interest Statement

The authors declare that the research was conducted in the absence of any commercial or financial relationships that could be construed as a potential conflict of interest.

## References

[1] Twitchell TE. (1951). The restoration of motor function following hemiplegia in man. Brain. 74:443–80. 10.1093/brain/74.4.443 14895765

[2] Perry J Burnfield J Gait Analysis: Normal and Pathological Function. Thorofare, NJ: Slack (1992).

[3] Dewald JP Pope PS Given JD Buchanan TS Rymer WZ. Abnormal muscle coactivation patterns during isometric torque generation at the elbow and shoulder in hemiparetic subjects. Brain. (1995) 118:495–510. 10.1093/brain/118.2.495 7735890

[4] Cruz TH Dhaher YY. Evidence of abnormal lower-limb torque coupling after stroke an isometric study. Stroke. (2008) 39:139–47. 10.1161/STROKEAHA.107.492413 18063824 PMC3641752

[5] Neckel ND Blonien N Nichols D Hidler J. Abnormal joint torque patterns exhibited by chronic stroke subjects while walking with a prescribed physiological gait pattern. J Neuroeng Rehabil. (2008) 5:19. 10.1186/1743-0003-5-19 18761735 PMC2553074

[6] Cruz TH Lewek MD Dhaher YY. Biomechanical impairments and gait adaptations post-stroke: multi-factorial associations. J Biomech. (2009) 42:1673–7. 10.1016/j.jbiomech.2009.04.015 19457488 PMC3641760

[7] Sakuma K Ohata K Izumi K Shiotsuka Y Yasui T Ibuki S. Relation between abnormal synergy and gait in patients after stroke. J Neuroeng Rehabil. (2014) 11:141. 10.1186/1743-0003-11-141 25257123 PMC4189205

[8] Finley JM Perreault EJ Dhaher YY. Stretch reflex coupling between the hip and knee: implications for impaired gait following stroke. Exp Brain Res. (2008) 188:529–40. 10.1007/s00221-008-1383-z 18446331 PMC2881696

[9] Marque P Simonetta-Moreau M Maupas E Roques C. Facilitation of transmission in heteronymous group II pathways in spastic hemiplegic patients. J Neurol Neurosurg Psychiatry. (2001) 70:36–42. 10.1136/jnnp.70.1.36 11118245 PMC1763478

[10] Maupas E Marque P Roques C Simonetta-Moreau M. Modulation of the transmission in group II heteronymous pathways by tizanidine in spastic hemiplegic patients. J Neurol Neurosurg Psychiatry. (2004) 75:130–5. 14707322 PMC1757451

[11] Dyer JO Maupas ES de Andrade Melo A Bourbonnais D Fleury J Forget R. Transmission in heteronymous spinal pathways is modified after stroke and related to motor incoordination. PLoS ONE. (2009) 4:e4123. 10.1371/journal.pone.0004123 19122816 PMC2607011

[12] Dyer JO Maupas ES de Andrade Melo A Bourbonnais D Forget R. Abnormal coactivation of knee and ankle extensors is related to changes in heteronymous spinal pathways after stroke. J Neuroeng Rehabil. (2011) 8:1. 10.1186/1743-0003-8-41 21806839 PMC3159134

[13] Hidler JM Carroll M Federovich EH. Strength and coordination in the paretic leg of individuals following acute stroke. IEEE Trans Neural Syst Rehabil Eng. (2007) 15:526–34. 10.1109/TNSRE.2007.907689 18198710

[14] Clark DJ Ting LH Zajac FE Neptune RR Kautz SA. Merging of healthy motor modules predicts reduced locomotor performance and muscle coordination complexity post-stroke. J Neurophysiol. (2009) 103:844–57. 10.1152/jn.00825.2009 20007501 PMC2822696

[15] Lauziere S Betschart M Aissaoui R Nadeau S Understanding spatial and temporal gait asymmetries in individuals post stroke. Int J Phys Med Rehabil. (2014) 2:2 10.4172/2329-9096.1000201

[16] Steele KM Rozumalski A Schwartz MH. Muscle synergies and complexity of neuromuscular control during gait in cerebral palsy. Dev Med Child Neurol. (2015) 57:1176–82. 10.1111/dmcn.12826 26084733 PMC4683117

[17] Shuman BR Schwartz MH Steele KM. Electromyography data processing impacts muscle synergies during gait for unimpaired children and children with cerebral palsy. Front Comput Neurosci. (2017) 11:50. 10.3389/fncom.2017.00050 28634449 PMC5460588

[18] Sulzer JS Roiz RA Peshkin MA Patton JL. A highly backdrivable, lightweight knee actuator for investigating gait in stroke. Robot IEEE Transact. (2009) 25:539–48. 10.1109/TRO.2009.2019788 22563305 PMC3342704

[19] Sulzer JS Gordon KE Dhaher YY Peshkin MA Patton JL. Preswing knee flexion assistance is coupled with hip abduction in people with stiff-knee gait after stroke. Stroke. (2010) 41:1709–14. 10.1161/STROKEAHA.110.586917 20576947 PMC3306800

[20] Akbas T Prajapati S Ziemnicki D Tamma P Gross S Sulzer J Hip circumduction is not a compensation for reduced knee flexion angle during gait. J Biomech. (2019). [Epub ahead of print]. 10.1016/j.jbiomech.2019.02.026 30876735

[21] Tan AQ Dhaher YY. Evaluation of lower limb cross planar kinetic connectivity signatures post-stroke. J Biomech. (2014) 47:949–56. 10.1016/j.jbiomech.2014.01.025 24556125 PMC4073298

[22] Allen JL Kautz SA Neptune RR. The influence of merged muscle excitation modules on post-stroke hemiparetic walking performance. Clin Biomech. (2013) 28:697–704. 10.1016/j.clinbiomech.2013.06.003 23830138 PMC3732538

[23] Zajac FE. Understanding muscle coordination of the human leg with dynamical simulations. J Biomech. (2002) 35:1011–8. 10.1016/S0021-9290(02)00046-5 12126660

[24] Zajac FE Neptune RR Kautz SA. Biomechanics and muscle coordination of human walking: part II: lessons from dynamical simulations and clinical implications. Gait Posture. (2003) 17:1–17. 10.1016/S0966-6362(02)00069-3 12535721

[25] Reinbolt JA Fox MD Arnold AS Õunpuu S Delp SL. Importance of preswing rectus femoris activity in stiff-knee gait. J Biomech. (2008) 41:2362–9. 10.1016/j.jbiomech.2008.05.030 18617180 PMC5507200

[26] Grood ES Suntay WJ Noyes FR Butler D. Biomechanics of the knee-extension exercise. Effect of cutting the anterior cruciate ligament. J Bone Joint Surg Am Vol. (1984) 66:725–34. 10.2106/00004623-198466050-00011 6725319

[27] Spoor C Van Leeuwen J. Knee muscle moment arms from MRI and from tendon travel. J Biomech. (1992) 25:201–6. 10.1016/0021-9290(92)90276-7 1733995

[28] Arnold AS Salinas S Hakawa DJ Delp SL. Accuracy of muscle moment arms estimated from MRI-based musculoskeletal models of the lower extremity. Comp Aided Surg. (2000) 5:108–19. 10.3109/10929080009148877 10862133

[29] van der Krogt MM Bar-On L Kindt T Desloovere K Harlaar J Neuro-musculoskeletal simulation of instrumented contracture and spasticity assessment in children with cerebral palsy. J Neuroeng Rehabil. (2016) 13:64 10.1186/s12984-016-0170-5 27423898 PMC4947289

[30] Arnold EM Delp SL. Fibre operating lengths of human lower limb muscles during walking. Philos Trans R Soc B Biol Sci. (2011) 366:1530–9. 10.1098/rstb.2010.0345 21502124 PMC3130447

[31] Arnold EM Hamner SR Seth A Millard M Delp SL. How muscle fiber lengths and velocities affect muscle force generation as humans walk and run at different speeds. J Exp Biol. (2013) 216:2150–60. 10.1242/jeb.075697 23470656 PMC3656509

[32] Mrachacz-Kersting N Lavoie B Andersen JB Sinkjaer T. Characterisation of the quadriceps stretch reflex during the transition from swing to stance phase of human walking. Exp Brain Res. (2004) 159:108–22. 10.1007/s00221-004-1941-y 15221163

[33] Delp SL Anderson FC Arnold AS Loan P Habib A Thelen DG. OpenSim: open-source software to create and analyze dynamic simulations of movement. Biomed Eng IEEE Trans. (2007) 54:1940–50. 10.1109/TBME.2007.901024 18018689

[34] Akbas T Sulzer J Implementing a virtual gait assistance device within a musculoskeletal simulation framework. In: 39th Annual Meeting of the American Society of Biomechanics. Columbus, OH (2015).

[35] Hicks JL Uchida TK Seth A Rajagopal A Delp SL. Is my model good enough? Best practices for verification and validation of musculoskeletal models and simulations of movement. J Biomech Eng. (2015) 137:020905. 10.1115/1.4029304 25474098 PMC4321112

[36] Pierrot-Deseilligny E Burke D The Circuitry of the Human Spinal Cord: Its Role in Motor Control and Movement Disorders. Cambridge, UK: Cambridge University Press (2005). 10.1017/CBO9780511545047

[37] Reinkensmeyer D Wynne J Harkema S A robotic tool for studying locomotor adaptation and rehabilitation. In: Engineering in Medicine and Biology, 2002. 24th Annual Conference and the Annual Fall Meeting of the Biomedical Engineering Society EMBS/BMES Conference, 2002. Houston, TX: Proceedings of the Second Joint, IEEE (2002).

[38] R Development Core Team. R: A Language and Environment for Statistical Computing. Vienna (2013). 30628467

[39] Brysbaert M Stevens M Power analysis and effect size in mixed effects models: a tutorial. J Cogn. (2018). p. 1–20. 10.5334/joc.10 PMC6646942 31517183

[40] Kerrigan DC Gronley J Perry J. Stiff-legged gait in spastic paresis. A study of quadriceps and hamstrings muscle activity. Am J Phys Med Rehabil. (1991) 70:294–300. 10.1097/00002060-199112000-00003 1741998

[41] Goldberg SR Anderson FC Pandy MG Delp SL. Muscles that influence knee flexion velocity in double support: implications for stiff-knee gait. J Biomech. (2004) 37:1189–96. 10.1016/j.jbiomech.2003.12.005 15212924

[42] Lewek MD Hornby TG Dhaher YY Schmit BD. Prolonged quadriceps activity following imposed hip extension: a neurophysiological mechanism for stiff-knee gait? J Neurophysiol. (2007) 98:3153–62. 10.1152/jn.00726.2007 17898135 PMC3293654

[43] Hesse S Bertelt C Jahnke M Schaffrin A Baake P Malezic M. Treadmill training with partial body weight support compared with physiotherapy in nonambulatory hemiparetic patients. Stroke. (1995) 26:976–81. 10.1161/01.STR.26.6.976 7762049

[44] Mayr A Kofler M Quirbach E Matzak H Fröhlich K Saltuari L. Prospective, blinded, randomized crossover study of gait rehabilitation in stroke patients using the Lokomat gait orthosis. Neurorehabil Neural Repair. (2007) 21:307–14. 10.1177/1545968307300697 17476001

[45] Dietz V Berger W. Interlimb coordination of posture in patients with spastic paresis. Brain. (1984) 107:965–78. 10.1093/brain/107.3.965 6478185

[46] Corna S Grasso M Nardone A Schieppati M. Selective depression of medium-latency leg and foot muscle responses to stretch by an alpha 2-agonist in humans. J Physiol. (1995) 484(Pt 3):803. 10.1113/jphysiol.1995.sp020705 7623294 PMC1157962

[47] Menegaldo LL de Toledo Fleury A Weber HI. Moment arms and musculotendon lengths estimation for a three-dimensional lower-limb model. J Biomech. (2004) 37:1447–53. 10.1016/j.jbiomech.2003.12.017 15275854

[48] Thelen DG Anderson FC. Using computed muscle control to generate forward dynamic simulations of human walking from experimental data. J Biomech. (2006) 39:1107–15. 10.1016/j.jbiomech.2005.02.010 16023125

[49] Sandercock TG Heckman C. Force from cat soleus muscle during imposed locomotor-like movements: experimental data versus Hill-type model predictions. J Neurophysiol. (1997) 77:1538–52. 10.1152/jn.1997.77.3.1538 9084618

[50] Dick TJ Biewener AA Wakeling JM. Comparison of human gastrocnemius forces predicted by Hill-type muscle models and estimated from ultrasound images. J Exp Biol. (2017) 220:1643–53. 10.1242/jeb.154807 28202584 PMC5450802

[51] Lee SS Arnold ASM de Boef Miara AA Wakeling JM. Accuracy of gastrocnemius muscles forces in walking and running goats predicted by one-element and two-element Hill-type models. J Biomech. (2013) 46:2288–95. 10.1016/j.jbiomech.2013.06.001 23871235 PMC4339187

[52] Ward SR Eng CM Smallwood LH Lieber RL. Are current measurements of lower extremity muscle architecture accurate? Clin Orthop Related Res. (2009) 467:1074–82. 10.1007/s11999-008-0594-8 18972175 PMC2650051

[53] Gerus P Rao G Berton E. Subject-specific tendon-aponeurosis definition in Hill-type model predicts higher muscle forces in dynamic tasks. PLoS ONE. (2012) 7:e44406. 10.1371/journal.pone.0044406 22952973 PMC3430662

[54] Perreault EJ Heckman CJ Sandercock TG. Hill muscle model errors during movement are greatest within the physiologically relevant range of motor unit firing rates. J Biomech. (2003) 36:211–8. 10.1016/S0021-9290(02)00332-9 12547358

[55] DeMers MS Hicks JL Delp SL. Preparatory co-activation of the ankle muscles may prevent ankle inversion injuries. J Biomech. (2017) 52:17–23. 10.1016/j.jbiomech.2016.11.002 28057351 PMC5798431

[56] Van der Krogt M Seth A Steele K Bar-On L Desloovere K Harlaar J A model of muscle spasticity in opensim. Gait Posture. (2013) 38:S16 10.1016/j.gaitpost.2013.07.039

[57] Jansen K De Groote F Aerts W De Schutter J Duysens J Jonkers I. Altering length and velocity feedback during a neuro-musculoskeletal simulation of normal gait contributes to hemiparetic gait characteristics. J Neuroeng Rehabil. (2014) 11:78. 10.1186/1743-0003-11-78 24885302 PMC4030738

[58] Demircan E Khatib O Wheeler J Delp S. Reconstruction and EMG-informed control, simulation and analysis of human movement for athletics: Performance improvement and injury prevention. In: Conference Proceedings: Annual International Conference of the IEEE Engineering in Medicine and Biology Society. IEEE Engineering in Medicine and Biology Society. Annual Conference, NIH Public Access (2009). 19964175 10.1109/IEMBS.2009.5333148 PMC4479296

[59] Rajagopal A Dembia CL DeMers MS Delp DD Hicks JL Delp SL. Full-body musculoskeletal model for muscle-driven simulation of human gait. IEEE Trans Biomed Eng. (2016) 63:2068–79. 10.1109/TBME.2016.2586891 27392337 PMC5507211

