# Supplementary material for: Neuromusculoskeletal Simulation Reveals Abnormal Rectus Femoris-Gluteus Medius Coupling in Post-stroke Gait
Source: Front Neurol. 2019 Apr 2;10:301. doi: 10.3389/fneur.2019.00301 (PMC6454148; doi:10.3389/fneur.2019.00301)
Supplement: Supplementary file 1 [file Table_1.pdf]

Table S1. Correlations between RF_{vel} and simulated muscle activations of RF and between RF and GMed activations in people with SKG during the involuntary response period. The table shows the correlation coefficients and significance (* $p < 0.05$, ** $p < 0.01$, *** $p < 0.001$) between RF_{vel} and simulated RF (left) and, RF and GMed activations (right) during IR for each participant with SKG.

Subject	$r_{RF_{vel}\ RF}$	$r_{RF\ GMed}$
S1	0.41	0.31
S2	0.58	0.95
S3	0.95	0.48
S4	0.23	1.35
S5	0.38	0.75
S6	0.56	0.72
S7	0.33	0.1
S8	0.2	0.38
S9	1.08	1.61
