# Supplementary material for: Neuromusculoskeletal Simulation Reveals Abnormal Rectus Femoris-Gluteus Medius Coupling in Post-stroke Gait
Source: Front Neurol. 2019 Apr 2;10:301. doi: 10.3389/fneur.2019.00301 (PMC6454148; doi:10.3389/fneur.2019.00301)
Supplement: Supplementary file 2 [file Table_2.pdf]

Table S2. Maximum and average values of the residual forces (F_x, F_y, F_z) and moments (M_x, M_y, M_z) from reduced residual algorithm (RRA) tool and the corresponding reserved joint actuators with maximum torque values from computed muscle control (CMC) tool of OpenSim are shown in the table. The values are collected across all simulated for each participant from the region of the gait cycles used in data analysis. **The maximum values are within the range of good simulation based on OpenSim guidelines (J. Hicks, Seth, Hamner, & Demers, 2015) for each participant with post-stroke SKG.**

	Residual Forces (N)						Residual Moments (N*m/s)						Reserved Actuators (N*m/s)	
	F_x		F_y		F_z		M_x		M_y		M_z		Corresponding reserved joint actuator	Max.
	Avg.	Max.	Avg.	Max.	Avg.	Max.	Avg.	Max.	Avg.	Max.	Avg.	Max.		
S1	-.62	14.4	.35	8.23	1.7	11.4	-.14	26.5	-1.8	11.6	-2.4	28.5	Left ankle flexion/extension	22.9
S2	1.9	8.77	1.6	8.93	1.3	6.54	.38	11.5	2.6	10.7	1.6	26.9	Left ankle flexion/extension	19.2
S3	1.4	9.65	1.8	5.07	1.2	12.7	0.2	13.5	-1.1	12.9	-.05	20.0	Right knee flexion/extension	17.0
S4	.84	6.38	-.48	7.62	-1.2	5.18	-.44	17.7	-3.8	9.61	.02	35.7	Left ankle flexion/extension	16.9
S5	2.3	14.4	.14	13.0	-.21	10.8	-.58	18.7	-1.7	17.0	1.5	28.5	Left ankle flexion/extension	24.1
S6	2.1	6.06	1.4	14.1	-.61	6.70	-.19	24.4	-3.3	18.3	-1.1	26.0	Right knee flexion/extension	20.8
S7	2.2	12.4	-.33	6.74	-1.6	9.29	-.02	25.3	.72	19.5	.13	33.9	Left hip flexion/extension	22.7
S8	2.0	10.6	.25	6.33	.73	7.30	-1.1	29.1	-4.2	15.5	-2.8	30.9	Left ankle flexion/extension	26.6
S9	1.4	5.67	1.8	2.50	1.4	4.45	.20	27.9	-.14	18.7	-.22	25.0	Right knee flexion/extension	22.2
