# Supplementary material for: Neuromusculoskeletal Simulation Reveals Abnormal Rectus Femoris-Gluteus Medius Coupling in Post-stroke Gait
Source: Front Neurol. 2019 Apr 2;10:301. doi: 10.3389/fneur.2019.00301 (PMC6454148; doi:10.3389/fneur.2019.00301)
Supplement: Supplementary file 3 [file Image_1.pdf]

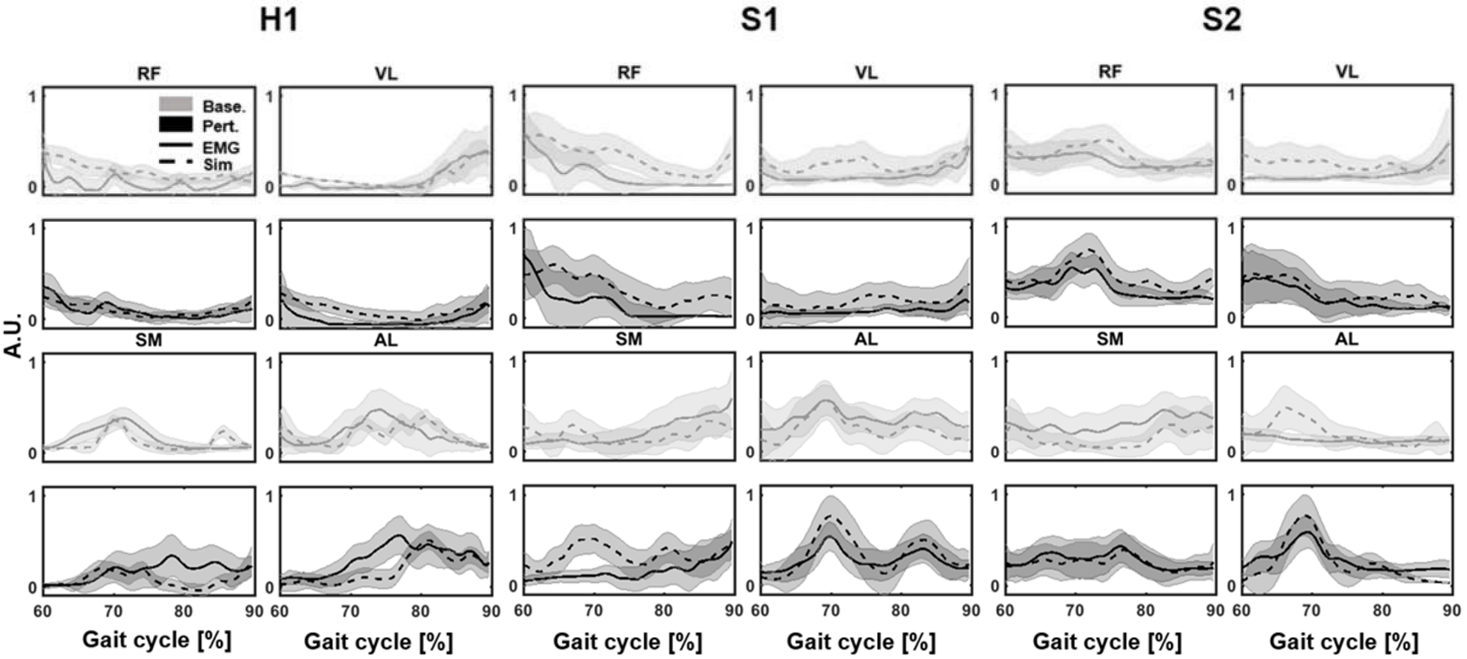

Figure S1: Validation of the simulated muscle activities (dashed lines) with integrated EMG activity (solid lines) for baseline (gray) and perturbation (black) for a representative healthy participant (H1) and two representative participants with post-stroke SKG (S1 and S2). The figure represents qualitative comparison between the simulated muscle activations and integrated EMG values for a healthy participant and two stroke participants with lowest and highest total Pearson correlation (baseline: $r_{S1} = .42$, $r_{S2} = .72$; perturbation: $r_{S1} = .46$, $r_{S2} = .76$), sum of the correlation values for baseline and perturbation, between RF EMG and simulated RF measures respectively during pre-swing and late-swing. EMG measures are collected from RF, vastus lateralis (VL), adductor longus (AL) and semimembranosus (SM). The healthy participant is in good agreement both for the baseline and perturbation conditions but there is a larger variance between participants and steps with post-stroke SKG variance observed by the different profiles and variance within participants indicated by shaded areas (± SE).
